# Identification of Ovarian Cancer Patients Most Likely to Achieve Chemotherapy Response Score 3 Following Neoadjuvant Chemotherapy: Development of a Predictive Nomogram

**DOI:** 10.3389/fonc.2020.560888

**Published:** 2020-10-05

**Authors:** Wei-feng Liang, Hui Li, Jie-ying Wu, Chang-hao Liu, Miao-fang Wu, Jing Li

**Affiliations:** ^1^Department of Gynecology and Obstetrics, Qilu Hospital (Qingdao), Cheeloo College of Medicine, Shandong University, Qingdao, China; ^2^Department of Gynecologic Oncology, Sun Yat-sen Memorial Hospital, Sun Yat-sen University, Guangzhou, China

**Keywords:** neoadjuvant chemotherapy, chemotherapy response score, nomogram, prognosis, ovarian cancer

## Abstract

**Background:** The chemotherapy response score (CRS) system is a reproducible prognostic tool for patients receiving neoadjuvant chemotherapy (NACT) for tubo-ovarian high-grade serous carcinoma (HGSC). Achieving CRS 3 following NACT can be used as a surrogate for progression-free survival (PFS) and overall survival (OS). This study aimed to identify predictors of CRS 3 and develop a predictive nomogram.

**Methods:** Data were extracted from 106 HGSC patients receiving NACT. Logistic regression was used to identify independent predictors for CRS 3. A nomogram was established based on the multivariate regression model.

**Results:** All patients received three cycles of NACT, and CRS 3 was observed in 24 (22.6%) patients. Compared with patients in the CRS 1–2 group, patients in the CRS 3 groups had significantly improved PFS (log-rank test *P* < 0.0001). The multivariate regression analysis identified post-NACT CA125, percent decrease in CA125, post-NACT human epididymis protein 4 (HE4), and post-NACT hemoglobin level as independent predictors of CRS 3. The Hosmer-Lemeshow test showed goodness-of-fit of this regression model (*P* = 0.272). The nomogram including these factors presented good discrimination (area under the curve = 0.82), good calibration (mean absolute error = 0.039), and a net benefit within the threshold probabilities of CRS 3 > 5%.

**Conclusions:** We validated the prognostic role of the CRS system and developed a nomogram that predicts the possibility of CRS 3 following NACT. The nomogram helps to identify patients who would benefit the most from NACT. More studies are warranted to validate this model.

## Introduction

Ovarian cancer is the most lethal of gynecologic cancer, with a 5-year overall survival (OS) of 45% (1). Primary debulking surgery (PDS) has been the standard treatment of advanced epithelial ovarian cancer. However, PDS can be associated with notable morbidity and even mortality, since aggressive surgical procedures may be needed to achieve no gross residual disease (R0 resection) (2). Therefore, for patients with high perioperative risk and patients with a low likelihood of achieving R0 resection in PDS, neoadjuvant chemotherapy (NACT) followed by interval debulking surgery (IDS) can be considered as an alternative option which has been recommended in the National Comprehensive Cancer Network (NCCN), International Federation of Gynecology and Obstetrics (FIGO) and European Society for Medical Oncology (ESMO) guidelines (2–4). Compared with PDS, NACT-IDS is less morbid and can increase the chances of achieving R0 resection (3). In addition, the use of NACT presents a unique opportunity to learn about tumor biology and evaluate drug sensitivity (5). ADDIN EN.CITE Despite these advantages, the utility of NACT is controversial. There is evidence that NACT can increase the risk of developing platinum-resistant disease (6, 7). In addition, even if NACT patients underwent R0 resection in IDS, their survival outcomes were not better than those of patients who had minimal residual disease after PDS (8). Therefore, a better selection of patients who are most likely to benefit from NACT is critical.

Recently, Böhm proposed a three-tiered chemotherapy response score (CRS) system based on the pathological examination of residual disease on omental specimens (9). Since its description, many studies have assessed the CRS independently and consistently validated it as a highly reproducible method to predict progression-free survival (PFS) and platinum sensitivity (10–15) for patients with tubo-ovarian high-grade serous carcinoma (HGSC). A recent meta-analysis by the HGSC CRS Collaborative Network confirmed that CRS 3 was significantly associated with improved OS (11). Based on the findings, the HGSC CRS Collaborative Network concluded that CRS can be considered as a surrogate for both PFS and OS (11). The International Collaboration on Cancer Reporting (ICCR) guidelines (16) and the ESMO-European Society of Gynaecologial Oncology (ESGO) consensus conference on ovarian cancer also recommend the scoring system as a reliable prognostic tool for HGSC patients who are treated with NACT (3).

Considering current evidence, we believe that the cohort of patients who achieve CRS 3 following NACT represents the clinical setting that gains the most from the NACT-IDS treatment modality. Previous studies reported that some routinely used clinical variables, such as CA125, HE4, and hemoglobin, have prognostic value for HGSC patients in the neoadjuvant setting (17–22). Given these findings, we hypothesized that these factors could also be used for early identification of patients with high likehood of achieving CRS 3. Hence, using data from two tertiary-referral university hospitals in China, we conducted a retrospective cohort study with the aim of identifying predictors of CRS 3 and developing a predictive nomogram for patient treated with NACT-IDS for HGSC.

## Patients and Methods

### Study Population

This study was approved by the Institutional Review Board of the Sun Yat-sen Memorial Hospital (SYSEC-KY-KS-2020-74). Patients who underwent NACT-IDS for International Federation of Gynecology and Obstetrics (FIGO) stages IIIC-IV ovarian, fallopian tube, or primary peritoneal HGSC between January 2012 and June 2019 were identified. We excluded all patients with borderline, germ cell and stromal tumors and patients who did not receive complete primary treatment. Demographic data, treatment notes and follow-up notes were reviewed. Serum levels of CA125 and human epididymis protein 4 (HE4) were measured at diagnosis, before each cycle of chemotherapy and at IDS, which was part of our routine clinical practice. Written informed consent was obtained from all patients.

The possibility of R0 resection in the primary debulking setting was assessed by a multidisciplinary team (MDT), which consisted of two experienced gynecological oncologists, one pathologist, and one radiologist. The first-line intravenous regimens for HGSC that were recommended in NCCN guidelines for ovarian cancer patients were used as neoadjuvant therapy (2). A minimum of six cycles of chemotherapy was recommended to all patients which included at least three cycles of adjuvant chemotherapy following IDS. Before NACT was initiated, histologic confirmation of diagnosis was obtained by fine needle aspiration, diagnostic laparoscopy or paracentesis. Patients who developed progressive disease during NACT were not submitted to IDS and were treated with second-line chemotherapy. They were not included in our analysis. For patients who responded to NACT or had a stable disease, IDS was performed after < 4 cycles of NACT. IDS was performed via a midline laparotomy with the maximum surgical effort to achieve R0 resection.

During the surveillance period, patients underwent a gynecologic examination and measurements of CA125 and HE4 every 3 months for the first 2 years, then every 6 months for the next 3 years, and every year thereafter. Imaging studies were performed at the discretion of the gynecologic oncologist.

### Pathological Evaluation and CRS

Specimens obtained during IDS were formalin-fixed and paraffin-embedded (16). The chemotherapy response was determined by two pathologists who were blinded to the clinical data, and the evaluation of all slides was unanimous. The CRS was assigned based on omental examination as described by Böhm (9). In brief, CRS 1 corresponds to no or minimal tumor response (no or minimal regression-associated fibroinflammatory changes limited to a few foci), including cases in which it is difficult to decide between regression and tumor-associated desmoplasia or inflammatory cell infiltration. CRS 2 means appreciable tumor response with viable tumor readily identifiable, ranging from multifocal, or diffuse regression-associated fibroinflammatory changes with viable tumor in sheets, streaks, or nodules to extensive regression-associated fibroinflammatory changes with multifocal residual tumor. CRS 3 corresponds to complete or near-complete response with no residual tumor or minimal irregularly scattered tumor foci seen as individual cells, cell groups, or nodules up to 2 mm maximum size. For cases with unanimous scores, the slides were re-evaluated, and consensus was reached by discussion.

### Statistical Analysis

The baseline characteristics of the patients are summarized as median and range for continuous variables and as the frequency and proportion for categorical variables. Student's *t*-test or the Mann-Whitney *U-*test was used to compare continuous variables, and the χ^2^ or Fisher's exact test was used to compare categorical variables as appropriate. Receiver operating characteristic (ROC) curves were constructed to assess the predictive value of CA125 and HE4 for CRS 3 following NACT. Optimal cut-off values were calculated by the maximum Youden indices. All factors associated with CRS 3 following NACT were evaluated with univariate logistic regression analysis, with results expressed as odds ratios (ORs) with 95% confidence intervals (CIs). Multivariate analysis with logistic regression test was performed to determine the independent predictors for CRS 3. The Hosmer-Lemeshow test was used to assess the goodness-of-fit of the logistic model, and a *P* < 0.05 indicated a poor agreement between the predicted probabilities and observed outcomes. A nomogram was developed based on the results of multivariate logistic regression analysis. The discriminative ability of the nomogram was assessed by Harrell's concordance index (C-index) and calibration. The C-index is an equivalent of the areas under the curve (AUC). An AUC of 0.6–0.7 was considered poor, 0.7–0.9 excellent, and >0.9 outstanding. A 1,000-sample bootstrapped calibration plot was developed, which was considered to have good performance when the calibration curve closely resembled the line representing perfect calibration [the prespecified acceptable mean absolute error (MAE) for the calibration curve was < 0.4]. Decision curve analysis (DCA) was conducted to evaluate the clinical benefit of the prediction model. PFS and OS were estimated using the Kaplan-Meier method and compared using the log-rank test. All statistical tests were two-sided, and *P* < 0.05 was considered significant. The statistical analysis was performed using STATA 12.0 (Stata Press, College Station, TX, USA), MedCalc 17.0 (MedCalc Software bvba, Ostend, Belgium) and R software version 3.6.3 (https://www.r-project.org).

## Results

### Demographic Characteristics of the Patients

A total of 106 patients were included in the analysis. Table 1 outlines the demographic characteristics. Before NACT, restricted activity was noted in 13 patients (12.3%); the reasons included massive ascites, pleural effusion and thromboembolic events. Anemia (hemoglobin < 100 g/l) was noted in 53 patients (50.0%). One hundred and one patients (95.3%) received carboplatin/paclitaxel every 3 weeks as the NACT regimen, while five (4.7%) patients were treated with weekly carboplatin/paclitaxel because of poor general status. Following NACT, anemia was noted in 29 patients (27.4%). All patients in our cohort received three cycles of NACT and underwent IDS within 3–4 weeks of the last cycle of NACT. On pathological evaluation of the omentum, the number of patients with CRS 1, CRS 2, and CRS 3 was 55 (51.9%), 27 (25.5%), and 24 (22.6%), respectively. The baseline characteristics before NACT were similar between the CRS 1–2 group and the CRS 3 group. After the completion of NACT, patients in the CRS 3 group had lower levels of CA125 (median: 24.9 vs. 37.6 U/ml; *P* = 0.002) and HE4 (median: 75 vs. 110 pmol/l; *P* = 0.002) than patients in the CRS 1–2 group. In addition, a higher percent reduction in CA125 (CA125 Pre-NACT—CA125 Post-NACT/CA125 Pre-NACT) was noted in the CRS 3 group than in the CRS 1–2 group (median: 98.4 vs. 96.0%; *P* < 0.001). An increase in the serum level of HE4 was observed in two patients, both of whom had an omental CRS of 1.

**Table 1:** Characteristics of patients.

**Variable**	**CRS 1-2 (*n =* 82)**	**CRS 3 (*n =* 24)**	***P*-value**
Age (years), median (range)	57 (37–71)	55 (47–78)	0.785
BMI (kg/m^2^), median (range)	22.3 (19.0–27.1)	22.1 (19.0–25.4)	0.567
FIGO stage, n (%)			
IIIC	75 (91.5)	19 (79.2)	0.137
IV	7 (8.5)	5 (20.8)	
ECOG performance status, n (%)			
Normal activity	71 (86.6)	22 (91.7)	0.488
Restricted activity	11 (13.4)	2 (8.3)	
R0 resection, n (%)			
Yes	63 (76.8)	24 (100)	<0.0001
No	19 (23.2)	0	
ICU stay following IDS, (%)			
Yes	2 (2.4)	2 (8.3)	0.222
No	80 (97.6)	22 (91.7)	
NACT regimen, (%)			
Three-week carboplatin/paclitaxel	78 (95.1)	23 (95.8)	0.883
Weekly carboplatin/paclitaxel	4 (4.9)	1 (4.7)	
CA125 (U/ml), median (range)			
Pre-NACT	1259.2 (106.4–17354.0)	1344.6 (239.4–13519.0)	0.207
Post-NACT	37.6 (7.4–2292.0)	24.9 (8.9–70.0)	0.002
Percent decrease (CA125 Pre-NACT—CA125 Post-NACT/CA125 Pre-NACT), (%) median (range)	96.0 (29.1–99.8)	98.4 (95.2–99.9)	0.000
HE4 (pmol/l), median (range)			
Pre-NACT	665 (105–6,397)	780 (141–1,500)	0.994
Post-NACT	110 (19–1,879)	75 (49–350)	0.001
Percent decrease (HE4 Pre-NACT—HE4 Post-NACT/HE4 Pre-NACT), (%) median (range)	80.1 (−88.0–98.7)	85.6 (37.0–96.7)	0.094
HGB (g/l), median (range)			
Pre-NACT	97.5 (69–120)	101.5 (81–121)	0.948
Post-NACT	103 (85–123)	111 (97–125)	1.000
Albumin (g/l), median (range)			
Pre-NACT	25 (16–37)	24 (20–27)	0.105
Post-NACT	33 (27–41)	32 (26–39)	0.171

*BMI, body mass index; CRS, chemotherapy response score; ECOG, eastern cooperative oncology group; FIGO, The International Federation of Gynecology and Obstetrics; HE4, Human epididymis protein 4; HGB, hemoglobin; ICU, intensive care unit; IDS, interval debulking surgery; NACT, neoadjuvant chemotherapy*.

The median follow-up duration for the entire cohort was 26.0 months (95% CI 20.4–33.6 months). The median PFS was 13.0 months (95% CI 12.0–15.0 months) for the CRS 1–2 group and 22.0 months (95% CI 19.0–24.0 months) for the CRS 3 group; the difference between the two survival curves (Supplementary Figure 1A) was statistically significant (log-rank test *P* < 0.0001). Median OS was not achieved. A comparison of the survival curves (Supplementary Figure 1B) showed a trend toward better OS in patients who achieved CRS 3 following NACT (log-rank test *P* = 0.132).

### The Predictive Ability and Cut-Off Values of Post-NACT CA125, Percent Decrease in CA125, Post-NACT HE4, and Percent Decrease in HE4

**Figure 1:**
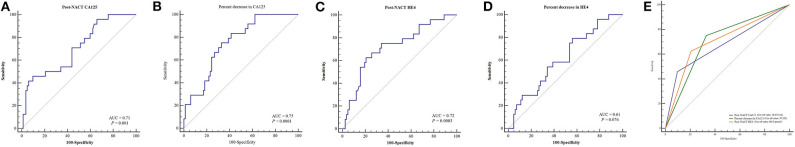
ROC curves to predict CRS 3 following NACT. **(A)** Post-NACT CA125. **(B)** Percent decrease in CA125. **(C)** Post-NACT HE4. **(D)** Percent decrease in HE4. **(E)** Comparison of AUCs. AUC, areas under the curve; CRS, chemotherapy response score; HE4, human epididymis protein 4; NACT, neoadjuvant chemotherapy; ROC, receiver operating characteristic.

Table 2 lists the AUC, optimal cut-off values, sensitivity, specificity, and Youden indices of post-NACT CA125, percent decrease in CA125, post-NACT HE4, and percent decrease in HE4 (HE4 Pre-NACT—HE4 Post-NACT/HE4 Pre-NACT) for predicting CRS 3 following NACT, and Figures 1A–D shows the ROC curves. No significant predictive value was noted for the percent decrease in HE4. The cut-off points based on the maximum value of the Youden index were 18.15 U/ml for post-NACT CA125, 97.32% for the percent decrease in CA125, and 80.15 pmol/l for post-NACT HE4. A further pairwise comparison of the AUCs was conducted (Figure 1E), which did not identify significant differences (total *P* = 0.892; AUC for post-NACT CA125: 0.68, 95% CI 0.57–0.79, *P* = 0.001; AUC for percent decrease in CA125: 0.71, 95% CI 0.60–0.82, *P* = 0.0001; AUC for post-NACT HE4: 0.71, 95% CI 0.61–0.81, *P* = 0.0002). Supplementary Figures 2A–C show the Kaplan-Meier survival graphs. Significant differences in PFS were identified when the cohort was categorized by post-NACT CA125 (log-rank test *P* = 0.028) and the percent decrease in CA125 (log-rank test *P* = 0.009), while the difference between the post-NACT HE4 groups did not reach statistical significance (log-rank test *P* = 0.343).

**Table 2:** Cut-off points to predict chemotherapy response score 3 following neoadjuvant chemotherapy.

	**AUC (95% CI)**	***P*-value**	**Cut-off value**	**Sensitivity (%)**	**Specificity (%)**	**Youden index (%)**
Post-NACT CA125	0.71 (0.61–0.79)	0.001	18.15	45.83	90.24	36.08
Percent decrease in CA125 (CA125 Pre-NACT—CA125 Post-NACT/CA125 Pre-NACT)	0.75 (0.66–0.83)	<0.0001	97.32%	75.00	67.07	42.07
Post-NACT HE4	0.72 (0.62–0.80)	0.000	80.15	62.50	79.27	41.77
Percent decrease in HE4 (HE4 Pre-NACT—HE4 Post-NACT/HE4 Pre-NACT)	0.61 (0.51–0.71)	0.076	76.46%	79.17	43.90	23.07

*AUC, areas under the curve; CI, confidence interval; HE4, human epididymis protein 4; NACT, neoadjuvant chemotherapy*.

### Predictors of CRS 3, Development of a Predictive Nomogram, and Clinical Application of the Nomogram

**Figure 2:**
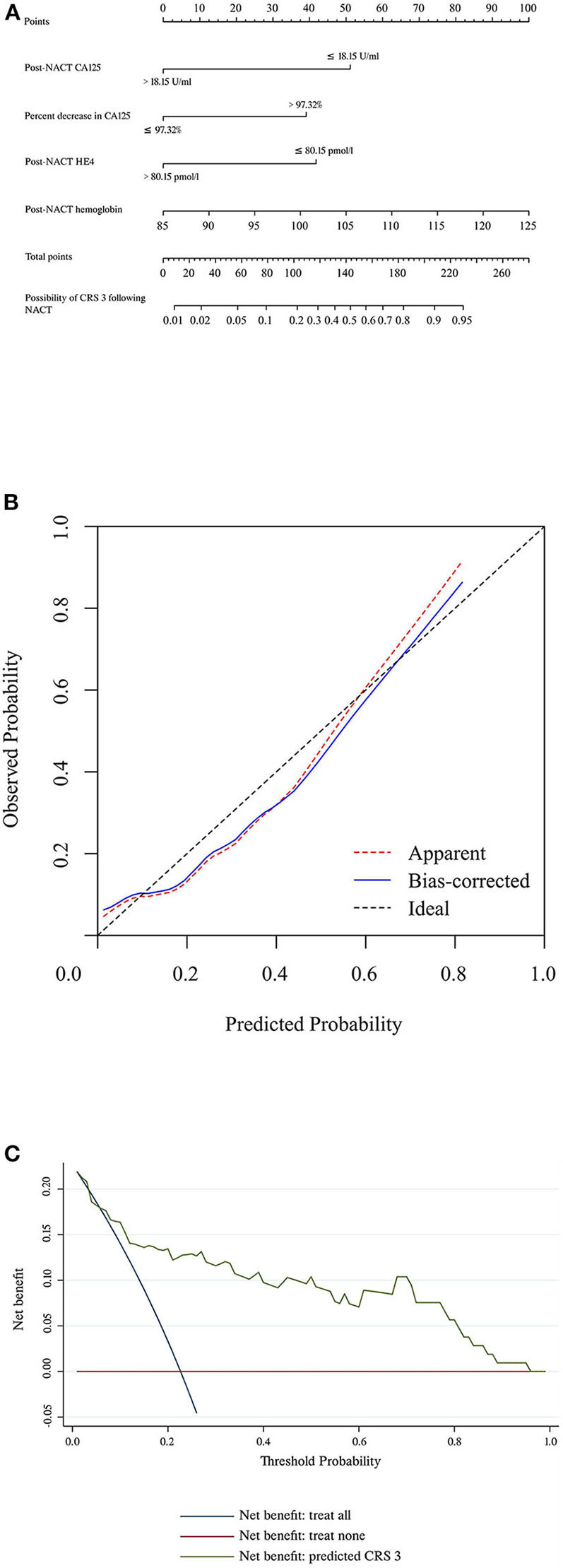
**(A)** Nomogram to predict the possibility of achieving CRS 3 following NACT. **(B)** Calibration plot for the nomogram. **(C)** Results of decision curve analysis. CRS, chemotherapy response score; HE4, human epididymis protein 4; NACT, neoadjuvant chemotherapy.

Table 3 summarizes the results of the univariate and multivariate regression analyses. Post-NACT CA125, percent decrease in CA125, post-NACT HE4, and post-NACT hemoglobin level were identified as independent predictors of CRS 3. The corresponding logistic regression equation is as follows: logit (CRS 3) = −8.95909–1.74322 ^*^ post-NACT CA125 + 1.33206 ^*^ percent decrease in CA125-−1.42433 ^*^ post-NACT HE4 + 0.08520 ^*^ post-NACT hemoglobin level. The Hosmer-Lemeshow test showed goodness-of-fit of this multivariate regression model (*P* = 0.272). Figure 2A shows the predictive nomogram derived from the β coefficients of the four independent factors. In ROC analysis, the AUC value of this nomogram was 0.85 (95% CI 0.75–0.95). Figure 2B shows the calibration plot, which suggests a favorable agreement between the predictions and observations (MAE = 0.039). Figure 2C shows the decision curve for the predictive nomogram. DCA demonstrated that the nomogram provided a higher net benefit than the “investigate all” (line crossing the X axis at 0.22–0.23) and “investigate none” (horizontal line at 0) strategies when the threshold probability of CRS 3 was > 5%.

**Table 3:** Logistic regression analysis of factors predicting chemotherapy response score 3 following neoadjuvant chemotherapy.

	**Univariate analysis**			**Multivariate analysis**		
**Variable**	**OR**	**95% CI**	***P*-value**	**OR**	**95% CI**	***P*-value**
Age (years)	1.03	0.96–1.10	0.427			
BMI (kg/m^2^)	1.02	0.80–1.31	0.864			
FIGO stage (III vs. IV)	2.82	0.81–9.87	0.105			
NACT regimen (3-week carboplatin/paclitaxel vs. weekly carboplatin/paclitaxel)	0.85	0.90–7.96	0.885			
CA125 (U/ml)						
Post-NACT (> 18.15 vs. ≤ 18.15)	0.13	0.04–0.38	0.000	0.17	0.05–0.64	0.008
Percent decrease (>97.32 vs. ≤ 97.32%)	6.11	2.18–17.16	0.001	3.79	1.15–12.54	0.029
Post-NACT HE4 (> 80.15 vs. ≤ 80.15pmol/l)	0.16	0.06–0.42	0.000	0.24	0.08–0.77	0.016
Albumin (g/l)						
Pre-NACT	0.93	0.81–1.07	0.329			
Post-NACT	0.93	0.80–1.08	0.340			
HGB (g/l)						
Pre-NACT	1.03	0.99–1.07	0.107			
Post-NACT	1.11	1.04–1.18	0.001	1.09	1.01–1.17	0.020

*BMI, body mass index; CI, confidence interval; FIGO, The International Federation of Gynecology and Obstetrics; HE4, human epididymis protein 4; HGB, hemoglobin; NACT, neoadjuvant chemotherapy; OR, odds ratio*.

Table 4 details the temporary cut-off values that were established to calculate the predictive performance at each cut-off. When the cut-off value was set at <3.0% for the predicted rate of CRS 3, none of the patients achieved CRS 3 following NACT. When the cut-off was ≥ 80.0%, a 100% rate of CRS 3 was observed.

**Table 4:** The possibility of chemotherapy response score 3 by nomogram prediction and actual rate in patients treated with neoadjuvant chemotherapy.

**Predicted CRS3 rate (%)**	**Actual CRS3 rate**
<3.0	0.0%
≥ 3.0– <10.0	9.1%
≥ 10.0– <20.0	12.5%
≥ 20.0– <30.0	20.0%
≥ 30.0– <40.0	33.3%
≥ 40.0– <80.0	56.3%
≥ 80.0	100.0%

*CRS, chemotherapy response score*.

## Discussion

Since the European Organization for Research and Treatment of Cancer (EORTC)-National Cancer Institute of Canada (NCIC) trial, the utility of NACT in the management of ovarian cancer patients has been increasing (23). There are three randomized controlled trials (RCTs) reporting that NACT-IDS is non-inferior to PDS with respect to survival outcomes (23–25). In addition, compared with PDS, NACT can increase the R0 resection rate and decrease the risks of post-operative morbidity and death (26). Based on these findings, many practice guidelines have recommended NACT-IDS as a reasonable option for ovarian cancer patients with advanced disease (2–4, 26). However, all the advantages associated with NACT do not translate into survival benefit. Most of the trials that support the use of NACT have been criticized since the reported PFS and OS were inferior to those reported in American ovarian cancer patients who were treated with PDS (5). Of additional concern is that the survival non-inferiority of NACT was not confirmed in a recent RCT by the Japan Clinical Oncology Group (27). In addition, NACT is given to patients with a high tumor burden, which leads to the selection of resistant tumor cells (28, 29). This observation has been confirmed in clinical studies where patients receiving NACT had a higher incidence of platinum-resistant recurrence at the first relapse than patients undergoing PDS (7). Given the current evidence, we believe that careful consideration should be given to the indication of NACT, and the identification of patients who can benefit the most from NACT is necessary.

The CRS system is the only method that is recommended in ESMO-ESGO guidelines for ovarian cancer patients to predict PFS and OS following NACT-IDS (3). Its reproducibility and accuracy have been validated in numerous studies (10–13, 15). The omental response to NACT that was graded with the scoring system was reported to be even more important than debulking status for the prognosis of patients. In line with these findings, the current study showed that patients who achieved CRS 3 following NACT had significantly improved PFS compared with patients who achieved CRS 1–2. Moreover, we identified independent predictors for CRS 3 and established a nomogram. The AUC value of this predictive model was 0.85; the calibration plot with an additional 1,000 bootstraps showed that the predicted probability of CRS 3 corresponded closely with the actual probability of CRS 3, which was further confirmed by the MAE value (0.039). The nomogram also showed excellent DCA results, with a net benefit when the probability of CRS 3 > 5%. Collectively, these findings suggested that the predictive nomogram had excellent discriminative ability and general applicability.

The number of administered NACT cycles in the present study did not exceed four, which is in line with the practical guidelines form American Society of Clinical Oncology (ASCO) and Society of Gynecologic Oncology (SGO) (26). There is evidence that the increasing number of NACT cycles has a negative prognostic influence. Each additional cycle of NACT was associated with a 4-month decrease in OS (30). Since this association was not significant among ovarian cancer patients who underwent IDS before the fourth cycle of NACT (30), the appropriate assessment of tumor response to the chemotherapy following the third cycle of NACT is necessary, which helps to determine whether additional cycles of NACT are needed. We noted that the predicted possibilities of achieving CRS 3 based on the nomogram were similar to the actual rates in our cohort of patients with <3% CRS 3 and patients with ≥ 80% CRS 3 (Table 4). The former group represents patients who do not respond well to NACT. Therefore, the fourth cycle of chemotherapy or a switch to the second-line chemo-regimens may be warranted, and close monitoring of the efficacy of subsequent chemotherapy is needed. On the other hand, patients achieving CRS 3 represent the cohort who have benefited the most from NACT and should be considered for IDS. Therefore, our predictive nomogram aids in therapeutic decision making.

Four independent predictors including post-NACT CA125, percent decrease in CA125, post-NACT HE4, and post-NACT hemoglobin level were used to develop the current nomogram. They are all inexpensive, convenient and routinely available biomarkers, so the nomogram can be readily used in resource-limited settings. CA125 and HE4 are the most commonly used tumor markers for the diagnosis and surveillance of HGSC patients. In the NACT setting, the predictive values of post-NACT CA125, and percent decrease in CA125 have been assessed previously (17–19, 31, 32). Le et al. (19) reported that patients with CA125 ≥ 35 U/ml following NACT were more likely to develop early relapse and death; other researchers have also reported similar results (17, 18). In Zeng's study, CA125 levels ≥ 200 U/ml before IDS were found to be associated with an increased risk of platinum-resistant recurrence (32). Pelissier et al. (31) showed that OS and PFS were significantly different between patients with a CA125 level ≤ 75 U/ml after the third cycle of NACT and those with a CA125 level > 75 U/ml. CA125 following NACT reflect the residual tumor burden, and the cut-off points used in the current study were selected to identify patients who had the minimum amount of residual disease (33). This explains why we selected a much lower cut-off value for post-NACT CA125 and a higher cut-off value for percent decrease in CA125 than those reported in previous studies (17–19, 31, 32). On the other hand, Böhm et al. (9) came to a different conclusion indicating that CA125 response to NACT could not predict CRS. Of note, there was considerable heterogeneity in the neoadjuvant regimen in Böhm's study. Some patients received carboplatin monotherapy and some underwent intraperitoneal chemotherapy. In addition, many patients received four or more cycles of NACT, but the authors did not clarify its influence on the possibility of achieving CRS3. For HE4, its prognostic role in HGSC patients receiving NACT was investigated in some retrospective studies involving small sample size. Pelissier et al. (20) reported that patients with HE4 > 115 pmol/l following the third cycle of NACT were more likely to develop platinum-resistant recurrence. However, many patients with low-grade disease were included in the analysis. Vallius et al. (21) reported that HE4 change >80% during the NACT period was associated with a prolonged OS. However, this study included only 25 HGSC patients, and some patients received more than three cycles of NACT. Although Plotti et al. (34) reported that the use of HE4 in combination with CA125 and computed tomography (CT) could predict R0 resection in IDS and prognosis of ovarian cancer patients receiving NACT, 43.9% of the 114 patients had clear cell carcinoma whose biological characteristics are significantly different from HGSC (35). Additionally, the authors neither provided data on recurrence or death nor conducted survival analysis. In view of these limitations, we believe that more studies are warranted to further explore the prognostic role of HE4 in the neoadjuvant setting. Interestingly, we found that the hemoglobin levels following NACT were independently associated with CRS 3. The possibility of CRS 3 increased 8.52% for a 1 g/l increase in the post-NACT hemoglobin level. A decreased level of hemoglobin can lead to reduced oxygen carrying capacity of the blood and finally induces tumor hypoxia which can make tumors resistant to chemotherapy (36–38). A low hemoglobin level before and during platinum-based chemotherapy has been observed to correlate with poor prognosis in patients with epithelial ovarian cancer, primarily due to missed and reduced chemotherapy doses (22, 39, 40). Given these findings and our results, we believe that correction of the anemia during NACT may be an important way to improve the clinical outcomes of these patients.

The following limitations of the current study should be stressed. First is the limited sample size. In NCCN, ESMO, and FIGO guidelines, PDS is recommended as the first-line treatment for ovarian cancer patients (2–4). In tertiary-referral university hospitals in China, including our institutions, the adherence to guideline recommendations is considered as the basic principle for the management of gynecologic cancer patients. Therefore, we only prescribe NACT to carefully selected patients. Although we pooled 8 years of data, the sample size of this work is only moderate, and it is difficult to conduct reliable internal and external validation. Second, because of the retrospective nature, missing data could not be avoided, and some potential predictors could not be included in the analysis. Third, although the PFS of our cohort was in line with that of previous reports, the follow-up period of our study was relatively short, and the median OS was not achieved. Finally, information about BRCA 1/2 status was not available in most of our patients, so its predictive role could not be assessed.

In conclusion, we identified predictors for achieving CRS 3 following NACT and developed a nomogram based on four routinely used clinical variables. Our preliminary results suggested that the nomogram has excellent predictive ability and general applicability. After further validation, we believe that this model could be utilized as an individualized tool to guide therapeutic selection for HGSC patients.

## Data Availability Statement

The raw data supporting the conclusions of this article will be made available by the authors, without undue reservation.

## Ethics Statement

The studies involving human participants were reviewed and approved by Institutional Review Board of the Sun Yat-sen Memorial Hospital. The patients/participants provided their written informed consent to participate in this study. The animal study was reviewed and approved by Institutional Review Board of the Sun Yat-sen Memorial Hospital.

## Author Contributions

WL and HL: data collection, data analysis, and development of methodology. JW and CL: data analysis and drafting the article. MW and JL: conception of the work and critical revision of the article. All authors contributed to the article and approved the submitted version.

## Conflict of Interest

The authors declare that the research was conducted in the absence of any commercial or financial relationships that could be construed as a potential conflict of interest.

## Supplementary Material

Supplementary Figure 1 Kaplan-Meier curves. **(A)** Progression-free survival (PFS). **(B)** Overall survival (OS). CRS, chemotherapy response score.

Supplementary Figure 2 Kaplan-Meier curves of progression-free survival. Patients were categorized by **(A)** Post-NACT CA125. **(B)** Percent decrease in CA125. **(C)** Post-NACT HE4. NACT, neoadjuvant chemotherapy.
