# Supplementary figures and images for: Identification of Ovarian Cancer Patients Most Likely to Achieve Chemotherapy Response Score 3 Following Neoadjuvant Chemotherapy: Development of a Predictive Nomogram
Source: Front Oncol. 2020 Oct 5;10:560888. doi: 10.3389/fonc.2020.560888 (PMC7571668; doi:10.3389/fonc.2020.560888)

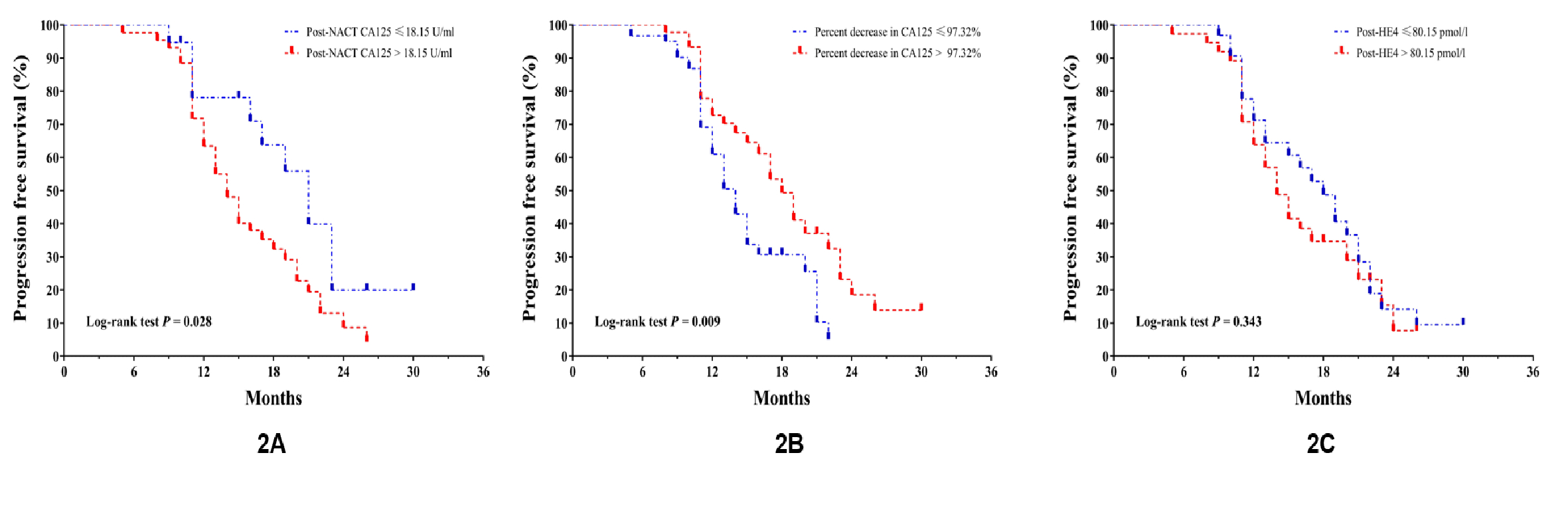

Supplement: Supplementary Figure 2 — Kaplan-Meier curves of progression-free survival. Patients were categorized by (A) Post-NACT CA125. (B) Percent decrease in CA125. (C) Post-NACT HE4. NACT, neoadjuvant chemotherapy. [file Image_2.jpg]
